# Awareness of Human Factors in the operating theatres during the COVID-19 pandemic

**DOI:** 10.1177/1750458920978858

**Published:** 2020-12-08

**Authors:** Carolina Relvas Britton, Gareth Hayman, Nicola Stroud

**Affiliations:** Cambridge University Hospitals, NHS Foundation Trust, Cambridge Biomedical Campus, Cambridge, UK

**Keywords:** Human Factors, Patient safety, Non-technical skills, Systems Resilience, COVID-19 pandemic, Operating Theatres, Healthcare, Communication, Teamwork

## Abstract

One of the priorities at our large Operating Theatres Department is to support awareness and basic education of the multi-disciplinary teams in clinical Human Factors, to help build competence and capacity in healthcare towards a resilient system. From May 2019 until February 2020, our Human Factors Champions embarked on a project called Observation of Non-technical Skills and Teamwork in the operating theatres (ONSeT), to monitor and evaluate the benefits of local Human Factors education. In September 2020, six months after the COVID-19 pandemic hit the UK and caused a major disruption of surgical services, we decided to investigate the usefulness of the project and the impact of COVID-19 in the operating theatres, looking through the eyes of the Human Factors Champions. Results pointed to a consensus about ONSeT having helped during the pandemic, with regards to how teams worked and in enabling team leaders to be more responsive. Human Factors Champions found that feedback on performance was received in a non-threatening way and observation of performance became ‘second nature’. As organisations need to develop critical thinking, we think that the ONSeT project has helped us build some capacity for this, from the front-line onwards.

**Provenance and Peer review:** Unsolicited contribution; Peer reviewed; Accepted for publication 14 November 2020.

## Introduction

### Background

More than 32,000 operations are performed each year at our large university teaching hospital, in the East of England. The large operating theatres department integrates 37 operating rooms and employs over 700 staff, delivering surgical care in all but one of the ten major surgical specialities (cardiothoracic surgery).

One of the department’s priorities is to support awareness and basic education of the multi-disciplinary teams in clinical Human Factors. This is rooted in the department’s overall patient safety strategy, as well as in the acknowledgement that Human Factors needs to be incorporated in the way we action improvement, by using a people-centred systems approach (the Chartered Institute of Ergonomics & Human Factors (CIEHF) and Health Education England (HEE) 2019).

Without access to a nationwide, focused and consistent approach (CIEHF & HEE 2019), our department has developed a project aimed at raising Human Factors awareness and provide basic multi-disciplinary Human Factors education in the operating theatres. This has been proposed as a fundamental step in building up Human Factors competence and capacity in healthcare towards a resilient system (CIEHF & HEE 2019).

During the year of 2019, we welcomed Human Factors expert guest lecturers presenting to our multidisciplinary teams, including one from the Association for Perioperative Practice (AfPP). We also ran Human Factors workshops and simulation sessions, and we gathered a group of operating theatres’ clinical leads to champion Human Factors in the department. In addition, to monitor and evaluate the benefits of Human Factors education, the Human Factors Champions embarked on a project called ‘Observation of Non-Technical Skills and Teamwork in theatres’ (ONSeT).

Through involvement on the ONSeT project, Human Factors Champions (nurses and operating department practitioners in the role of clinical leads) spent time observing the teams at work in the various operating theatres and applied two validated observation tools – the ‘Scrub Practitioners List of Intra-operative Non-technical skills’ (SPLINTS) (Mitchell et al 2012) and the ‘Mayo high performance teamwork scale’ (Malec et al 2007) – to capture and articulate how clinical teams’ performance could be enhanced.

### The ONSeT project

**Figure 1:**
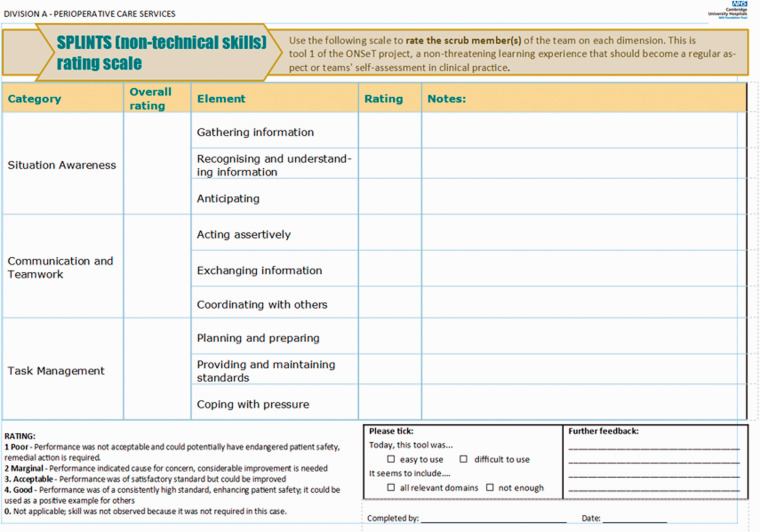
The SPLINTS tool observation sheet. Source: reproduced with permission from Elsevier (Mitchell et al 2012).

**Figure 2:**
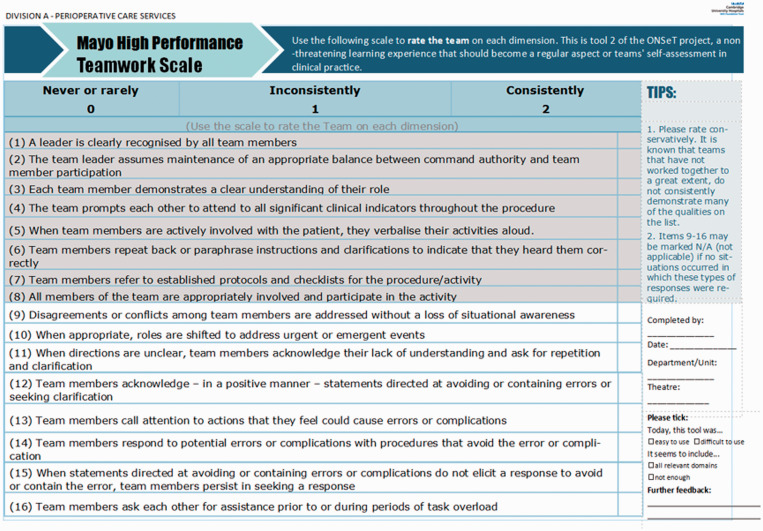
The ‘Mayo’ tool observation sheet. Source: reproduced with permission from Wolters Kluwer (Malec et al 2007).

The ONSeT project emphasised person-level human factors rather than system-level, as it is most common in critical care and operating theatres services where Human Factors training takes place in the NHS (NHS Institute for Innovation and Improvement 2010). Figures 1 and 2 depict the local observation sheets devised for the observation tools of SPLINTS and ‘Mayo’, respectively.

Team leads observed teams at work in the operating theatres, within their own units and other units, for an average of 4h per week. As rating systems, both tools provided lists of observable behaviours, a scale to score observed behaviours, and the opportunity to note relevant feedback. Team leads’ presence in the operating theatres, conducting ONSeT, was rapidly assimilated as part of their roles as clinical leads. As such, any potential assessment bias or awareness of being observed (Hawthorne effect) was not considered to impede the significance of this form of exercising clinical and educational leadership. Instead, ONSeT was seen as a tool to structure feedback to teams and team members, and to base discussions about ways of improving efficiency and safety in theatres.

Outputs were seamlessly collated (using a survey tool), reported centrally every week, and analysed by teams on a monthly basis. The ONSeT project ran from May 2019 until February 2020, and, during that time, 250 assessments were made.

### Impact of COVID-19

On 17 March 2020, as mandated by the NHS Chief Executive’s letter to all NHS organisations, our department ceased all non-cancer elective surgery and focused on deploying resources to safely carry out emergent operations, while also supporting critical care surge areas (Britton et al 2020). As a result, from March 2020, ONSeT data collection and regular meetings were interrupted due to the disruption of surgery, and of the department, by the COVID-19 pandemic.

## Aim and method

In September 2020, and in the context of resuming surgical work after the first peak of the pandemic, we decided to investigate: (1) whether the experience with the project helped during the pandemic in theatres, and (2) what could be said about the impact of COVID-19 thus far in the operating theatres, looking through the eyes of the Human Factors Champions.

As part of the ONSeT approved local audit protocol, we sent out a survey to all the Human Factors Champions, asking their opinions and suggestions on future Human Factors training in the department. We asked whether ONSeT had been useful and, though a combination of open and closed structured questions, explored the Champions’ ideas on the impact of the pandemic on each of the domains included in the tools, embracing all theatres, regarding the last six months.

## Results

### Perceived usefulness of the ONSeT project

**Figure 3:**
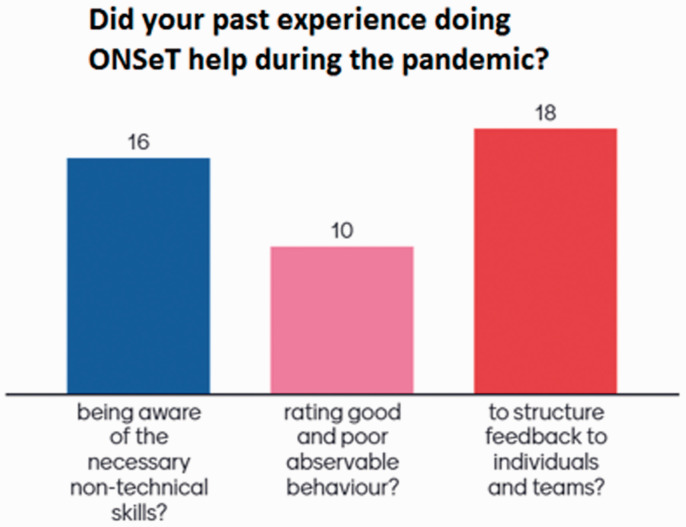
The usefulness of the ONSeT project ‘during the pandemic’ period (March 2020 to September 2020), as perceived by 22 participants.

There was consensus about the ONSeT experience having helped during the pandemic, as shown in Figure 3 and in the excerpts to follow not only with regards to how teams worked, but also in having enabled team leaders to be more responsive to the needs of their teams. In addition, participants agreed that ONSeT helped measure if teams were clearly committed to tasks (82%) and if there was a high degree of trust (73%). Fewer participants agreed that it also helped measure if teams were focused on results (36%), if members held one another accountable (55%), and whether there was constructive engagement in conflict situations (36%).

Finally, on a scale from one (strongly disagree) to five (strongly agree), Human Factors Champions evaluated how better they, as team leads, were able to help maintain safety in theatres (weighted average of 3.7), to assess and give feedback to teams (3.6 weighted average) and to be trusted as a leader (3.3 weighted average). Accordingly, they agreed that the ONSeT project had directly helped all teams maintain safe practice, self-assess and look to improve, and to participate (4.1 weighted average).

*The framework from the ONSeT process meant that the teams were used to receiving feedback in a non-threatening way which allowed for the transmission of information regarding issues without blame.*

*Communication, team work and being able to anticipate became very important skills during the pandemic. ONSeT highlights these skills.*

*Observing non-technical skills has become almost second nature which means I can pick up on cues that are not obvious.*

*Being aware of how the system impacts on our daily tasks and how actions enhance productivity and efficiency. Staff is aware of their impact on the task required to complete a list given the extra requirement for safety.*

*It is important to evaluate what we are doing as a team, assess standards are being maintained even with the significant changes that have taken place, to monitor if team dynamics are strong.*

*ONSeT is a good guide to my everyday work with my staff/team.*

### Experiences on the impact of COVID-19

**Figure 4:**
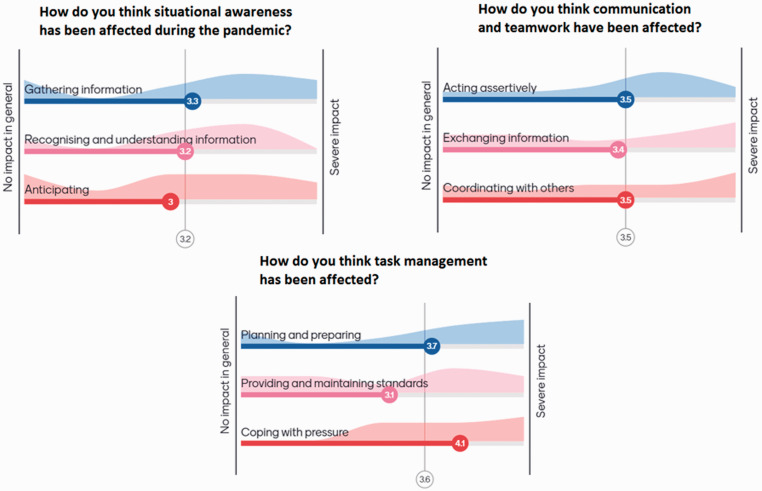
The impact on the domains included in the SPLINTS tool, on a scale from one (no impact in general) to five (severe impact) as perceived by 11 respondents

The impact of the pandemic on situation awareness, communication and teamwork, and task management (the three domains of the SPLINTS tool) was perceived to be of moderate degree, as seen in Figure 4 and excerpts from participants’ accounts below.

*Forward planning has become more important, which has improved anticipation.*

*Anticipation was very important due to nature of set up in theatres due to COVID… fewer staff and complexity of getting equipment into theatre once procedure had commenced.*

*I think a lot of information was given to the team, and on top of it things kept changing very often. There were conflicting ideas and also due to shortage of PPE eg, masks, this was a real problem.*

*Heightened anxiety due to COVID so more aware of importance of information given.*

*Acting assertively – I think improved, in the PPE* [protective personal equipment] *you had to be clear and concise to get your point across.*

*Sharing information was much harder, eg, hard to use phone in full PPE and verbal messages could get garbled.*

*The constant changes and inconsistent messages created the challenges.*

Interestingly, the statements around this were that forward planning had become more important (especially as it was harder to come out of theatres to get a bit of kit that was missing, for example). Constant change, however, made situational awareness harder to maintain, due to increased cognitive load. Assertiveness was believed to be enhanced, as a response mechanism to rapid changes in protocols, while coordinating with others was hindered by the use of respiratory protective equipment (such as filtered masks and hoods). Concerning task management, comments referred to the system and the organisation levels but focused greatly on ‘coping with pressure’ as it increased significantly.

**Figure 5:**
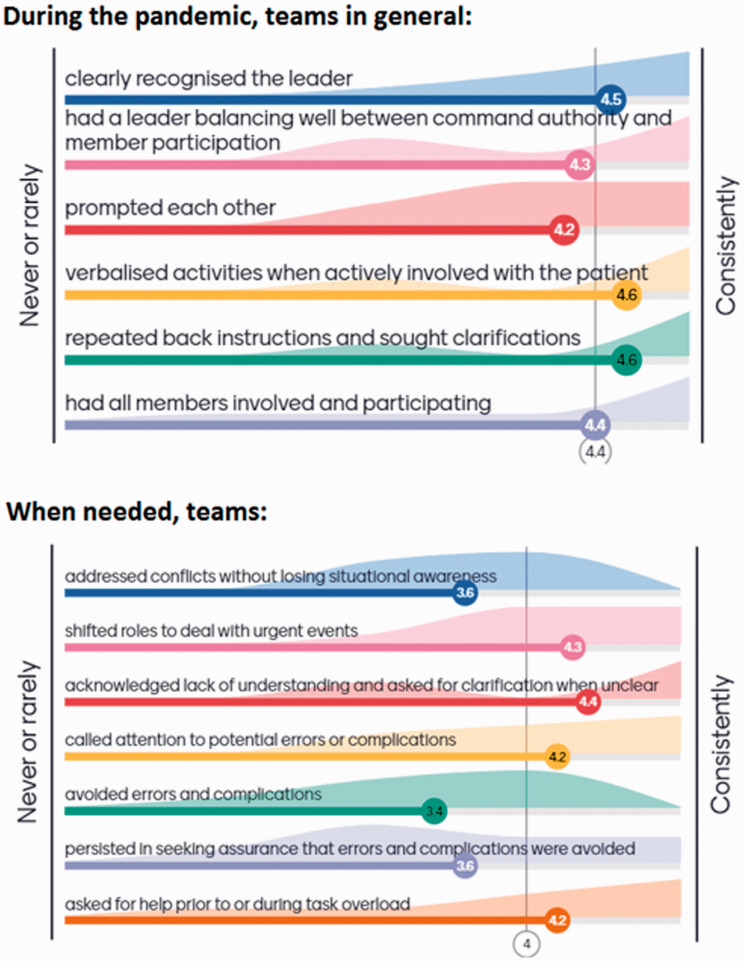
Assessment of teams’ performance during the first six months of the pandemic, in the domains included in the ‘Mayo’ tool, on a scale from 1 (never or rarely) to 5 (consistently) as perceived by ten respondents

Exploring the territories specifically mapped by the ‘Mayo’ tool, respondent Human Factors Champions tended to attribute high scores to teams, meaning that specific exemplar behaviours were frequently or consistently observed. This is shown in Figure 5, and in the following selected statements.

*Very important to have a recognised leader during difficult different and stressful times.*

*All members involved and clear leader – both improved because there were fewer people inside the theatre.*

*Leader balanced between leading/participating – most of the time the 'red hat'* [in-charge] *was now scrubbed as limited people in theatre. Not supposed to do before, but less of a problem because the staff weren't moving in and out, less change mid case.*

*I think constant information sharing with updates and practice changes helped a lot.*

*I ensure that patient and staff safety is our priority. I always ask if it is not clear to me, I always update myself and attend meeting with other senior staff for guidelines.*

*Staff wanted to be sure that they were doing the right thing and patient safety was ensured.*

*Teams will challenge conflict sometimes but will escalate if they feel unable to, staff have been reminded of PACE* [communication tool]*, and the importance of identifying errors or complications. Many staff have shifted roles to deal with urgent.*

## Discussion and conclusion

As part of the ongoing work in providing effective basic Human Factors education, our department initiated the ONSeT project in May 2019, capitalising on Human Factors Champions’ knowledge and participation as team leaders to promulgate and embed Human Factors awareness in the operating theatres. Six months after the start of the COVID-19 pandemic, which forced the department and staff to many new adaptations, the overall opinion was that the Human Factors training, and the ONSeT project in particular, had had a positive effect.

Human Factors Champions (clinical lead theatre nurses and operating department practitioners) felt better able to perform their roles as team leads, in supervising their teams and providing accurate timely feedback. This is evidenced by the granular detail and the particular experiential accounts that were shared when Champions were asked about the effect of the pandemic (effects related to extensive new protocols, new equipment, competing priorities, depleted teams, etc) in the domains they became very familiar with by doing ONSeT. These domains include ‘situational awareness’, ‘communication and teamwork’ and ‘task management’ (included in the SPLINTS tool), as well as leadership behaviours, safety culture and conflict resolutions (as evaluated by the ‘Mayo scale’).

Perhaps counter-intuitively, Champions felt that teamwork had suffered a positive impact, as, for example, member participation was improved by enhanced wellbeing conversations which, in turn, opened the way to more ‘outside of the box’ thinking, with ideas and creative solutions being shared very rapidly. The ability of teams and team members to adapt was generally perceived to have grown, and there was some focus on role augmentation and up-skilling. In addition, it was felt that not losing sight of patient safety, despite of and because of an increased cognitive load due to extensive new learning, became the very defining feature of all theatres’ work during the pandemic.

It was also interesting to find that statements explaining how forward planning had become more important were far more prevalent than the opposing perspective that planning ahead was made difficult by constant changes in practice and pathways.

By receiving Human Factors training and doing ONSeT, team leads became able to articulate challenges and barriers, using a shared mental model of the impact of Human Factors in individual and team performance in the operating theatres. This could have, arguably, expedited our adaptations at micro and meso levels, so that patient and staff safety were not seen as competing but synergetic priorities.

As the CIEHF pointed out in the recent publication on ‘Achieving sustainable change: capturing lessons from COVID-19’, organisation learning is paramount. If ‘the importance of resilience became very clear during COVID-19 where organisations underwent radical change affecting every area of care’, then it is imperative to engage in critical reflection at all levels of the system.

Furthermore, and at the tipping point of a second wave in the UK, it is crucial to consolidate and share all short-term learning significant to perioperative care services. This seems particularly important when an unprecedented backlog of elective surgery means that a number of patients waiting longer than 52 weeks for their operation increased 50-fold between February and June 2020 (NHS 2020).

As such, this small survey provides limited evidence that there are further relevant questions arising from reflecting about our adaptation and resilience as a department and as teams, namely on:

- What might have been the impact of the pandemic on teams, teamwork and on the way that people work together in theatres?
- What has the pandemic taught us about human factors’ impacting on theatres’ productivity and efficiency and patient safety in times of crisis?
- Which are the (tangible and less tangible) gains in raising awareness and capability in human factors among theatre staff? And what about when facing critical capacity-demand misalignment?

During and because of the COVID-19 pandemic, organisations need to develop critical thinking (CIEHF 2020), and so we think that ONSeT has helped us build some capacity for this, from the front-line onwards, which should be capitalised upon to better plan and deliver essential surgical care.

*No competing interests declared*
